# Studying Spatial Protein Quality Control, Proteopathies, and Aging Using Different Model Misfolding Proteins in *S. cerevisiae*

**DOI:** 10.3389/fnmol.2018.00249

**Published:** 2018-07-23

**Authors:** Kara L. Schneider, Thomas Nyström, Per O. Widlund

**Affiliations:** Department of Microbiology and Immunology, Institute of Biomedicine, Sahlgrenska Academy, University of Gothenburg, Gothenburg, Sweden

**Keywords:** protein quality control, spatial protein quality control, protein misfolding, misfolding model, inclusions, aging, cell stress, temperature-sensitive

## Abstract

Protein quality control (PQC) is critical to maintain a functioning proteome. Misfolded or toxic proteins are either refolded or degraded by a system of temporal quality control and can also be sequestered into aggregates or inclusions by a system of spatial quality control. Breakdown of this concerted PQC network with age leads to an increased risk for the onset of disease, particularly neurological disease. *Saccharomyces cerevisiae* has been used extensively to elucidate PQC pathways and general evolutionary conservation of the PQC machinery has led to the development of several useful *S. cerevisiae* models of human neurological diseases. Key to both of these types of studies has been the development of several different model misfolding proteins, which are used to challenge and monitor the PQC machinery. In this review, we summarize and compare the model misfolding proteins that have been used to specifically study spatial PQC in *S. cerevisiae*, as well as the misfolding proteins that have been shown to be subject to spatial quality control in *S. cerevisiae* models of human neurological diseases.

## Introduction

The presence of protein inclusions is a hallmark of many age-related neurological diseases (Kaytor and Warren, 1999; Koo et al., 1999; Paulson, 1999). There is much evidence to suggest that the misfolded proteins generated during progression of these diseases are deposited into inclusions by the cell’s protein quality control (PQC) machinery to shield cellular components from their toxic properties (Saudou et al., 1998; Arrasate et al., 2004; Takahashi et al., 2008; Tyedmers et al., 2010).

Though protein deposits are common to many neurological diseases, inclusions can also be seen in aged neuronal cells of healthy animals (Fiori, 1987; Peters et al., 1991), reinforcing the idea that inclusions are a normal response of the PQC machinery to misfolded proteins. However, there are several lines of evidence for an age-related decline in the cells ability to process damaged proteins, which may explain the increased incidence of neurological disease with age (Cuervo and Dice, 2000; Koga et al., 2011; Kruegel et al., 2011; Andersson et al., 2013; Oling et al., 2014; Saez and Vilchez, 2014).

While the observed inclusions vary in form and intracellular location across species ranging from bacteria to humans, the formation of cellular inclusions in response to aberrantly folded proteins is evolutionarily conserved and is a consequence of the cell’s ongoing effort to maintain protein homeostasis, or proteostasis. Organisms across the evolutionary tree have evolved systems of temporal quality control and spatial quality control to maintain a functioning proteome (Hartl and Hayer-Hartl, 2009; Mogk and Bukau, 2017). The chaperones of the temporal quality control system ensure proper folding of newly synthesized proteins, attempt to refold misfolded proteins and promote degradation of those that cannot be effectively refolded (Hill et al., 2017; Josefson et al., 2017; Sontag et al., 2017). A system of spatial quality control runs in parallel to sort and deposit potentially harmful misfolded proteins and its action is most apparent when the temporal quality control system fails or is overloaded. When this occurs, misfolded proteins are partitioned into inclusions which shield the cell from their toxicity and can aid in their eventual clearance (Taylor et al., 2003; Escusa-Toret et al., 2013; Wolfe et al., 2013).

Due to the general conservation of the PQC machinery, several model organisms have been used to study not only the pathways involved in PQC, but also how they manage misfolded proteins that have been identified as important in the development of several human age-related neurological diseases. One of these is the proven model eukaryote, *Saccharomyces cerevisiae*. It is well-suited for the study of PQC, particularly in the context of aging, because aging can be studied in two major contexts: non-proliferative and proliferative cells. Yeast can be grown to stationary phase, where they eventually lose viability. This loss of viability, sometimes called chronological aging, can in many ways mimic aging experienced by differentiated cells like neuronal cells. Furthermore, proliferative cells, like stem cells, can also be modeled since yeast similarly undergo asymmetric cell divisions.

Asymmetric divisions are important in the context of spatial quality control because they allow damage, notably damaged proteins, to be segregated asymmetrically. During asymmetric cell division in yeast, the daughter cell is rejuvenated and one reason for this is that damaged or misfolded proteins are retained in the mother cell as part of the system of spatial quality control (Aguilaniu et al., 2003; Shcheprova et al., 2008). Evidence is emerging that some stem cell types also segregate damage asymmetrically to allow the stem cell lineage to propagate free of damage (Rujano et al., 2006; Fuentealba et al., 2008; Bufalino et al., 2013; Ogrodnik et al., 2014). Since this asymmetrical division of damage is limited to proliferating cells, it is possible that the higher incidence of inclusions in differentiated cells like neurons may be a consequence of the inability of the spatial quality control machinery to remove damage through division. Spatial PQC, therefore, plays a key role in both aging and age-related neurological disease and yeast has been successfully used to study the pathways involved in both contexts.

A key reason for this success has been due to the development of model proteins that either probe how the cell responds to misfolding proteins in general or how they deal with those that are thought to be the main causative agents of human disease. Model misfolding proteins are especially useful in the study of both temporal and spatial quality control as they can be used to track processing by the quality control machinery with minimal perturbation to the system itself. Fluorescently tagged substrates are indispensable, particularly in the study of spatial quality control, as they allow straightforward tracking of aggregate formation and localization by light microscopy. They also allow the study of spatial quality control in relation to temporal control by following aggregates in the cell through time. Several misfolding model proteins have been developed for these purposes. Furthermore, several human disease proteins have also been successfully used in yeast to study both the nature of the toxicity of the misfolded proteins, as well as how the PQC machinery responds to these proteins. Herein, we review the major model proteins used in *S. cerevisiae* to study spatial PQC pathways and the role of spatial quality control in the molecular basis of human disease.

## Model Misfolding Proteins

**FIGURE 1:**
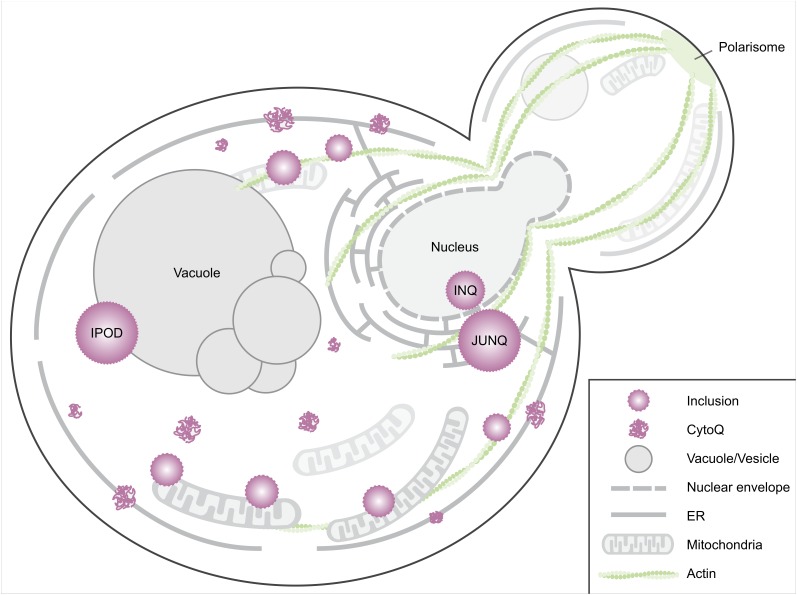
Spatial protein quality control sites in *Saccharomyces cerevisiae*. When induced to misfold, proteins aggregate in the cytosol and on the ER membrane (Escusa-Toret et al., 2013). These initial cytosolic aggregates are called CytoQ (Miller et al., 2015), stress foci (Spokoini et al., 2012), peripheral aggregates (Specht et al., 2011), cytosolic puncta (Kaganovich et al., 2008) or Q-bodies (Escusa-Toret et al., 2013). These coalesce into larger structures, usually referred to as inclusions. Smaller inclusions have been observed tethered to actin cables (Liu et al., 2010; Song et al., 2014) or are captured by mitochondria (Zhou et al., 2014; Böckler et al., 2017). Yeast cells have several distinct larger inclusions including, but not limited to, the intranuclear quality control site (INQ), the juxtanuclear quality control site (JUNQ), and the perivacuolar IPOD site (Kaganovich et al., 2008; Miller et al., 2015). Other IPOD-like peripheral inclusions likely exist as some aggregates of model substrates do not co-localize with the IPOD, e.g., the non-amyloidogenic disease protein OPTN (Kryndushkin et al., 2012). An additional site, the age-associated protein deposit site (APOD, not depicted here) has been identified in aged cells (Saarikangas and Barral, 2015).

Model misfolding proteins have helped to define the different quality control sites that have been identified in yeast (Kaganovich et al., 2008; Miller et al., 2015; Hill et al., 2017) (**Figure 1**). When induced, they often initially accumulate at stress foci, called CytoQs/Q-bodies/peripheral aggregates, in the cytoplasm or at the surface of organelles including the endoplasmic reticulum, mitochondria, and vacuole (Specht et al., 2011; Spokoini et al., 2012; Escusa-Toret et al., 2013; Miller et al., 2015). During prolonged stress, the aggregates coalesce into larger foci, often called inclusions, which are deposited or collected at several defined sites: the juxtanuclear quality control (JUNQ), the intranuclear quality control (INQ) and the insoluble protein deposit (IPOD) site (Kaganovich et al., 2008; Miller et al., 2015). They also can associate with, and be imported into, mitochondria (Zhou et al., 2014; Ruan et al., 2017). Other sites are likely to exist as some misfolding human disease models do not appear to localize to these defined sites (Tenreiro et al., 2014; Farrawell et al., 2015). The misfolding models are summarized in **Table 1**. We grouped them into three general categories: Temperature-sensitive (Ts) misfolding proteins, continuously misfolding proteins, and human disease proteins. For each category, we will first describe the development of model proteins for *S. cerevisiae* and then discuss how they have been used to elucidate spatial quality control pathways.

**Table 1:** Fluorescently tagged misfolding model proteins.

Name	Origin	Mutant model	Misfolding	Control	Expression	Fluorescent tag location	Reference
Luciferase	*P. pyralis*	Heat denaturation	Ts	N/A	Constitutive – ACT1	N-terminus	Specht et al., 2011
FlucSM	*P. pyralis*	Heat denaturation/missense	Ts	Fluc	Constitutive	C-terminus	Ruan et al., 2017
ubc9-2	*S. cerevisiae*	Heat denaturation/missense	Ts	UBC9	Induced – GAL	N-terminus	Kaganovich et al., 2008
guk1-7	*S. cerevisiae*	Heat denaturation/missense	Ts	GUK1	Constitutive – TDH3	C-terminus	Comyn et al., 2016
gus1-3	*S. cerevisiae*	Heat denaturation/missense	Ts	GUS1	Constitutive – TDH3	C-terminus	Comyn et al., 2016
pro3-1	*S. cerevisiae*	Heat denaturation/missense	Ts	PRO3	Constitutive – TDH3	C-terminus	Comyn et al., 2016
ugp1-3	*S. cerevisiae*	Heat denaturation/missense	Ts	UGP1	Constitutive – TDH3	C-terminus	Comyn et al., 2016
Actin(E364K)	*D. melanogaster*	Missense	Continuous	None available	Induced – GAL	C-terminus	Kaganovich et al., 2008
VHL	*H. sapiens*	Absent binding partner	Continuous	N/A	Induced – GAL	N-terminus	Kaganovich et al., 2008
ΔssCPY^∗^	*S. cerevisiae*	Missorting	Continuous	N/A	Constitutive – PRC1, Induced – GAL	C-terminus	Park et al., 2007, 2013
ΔssPrA	*S. cerevisiae*	Missorting	Continuous	N/A	Constitutive – TDH3	C-terminus	Prasad et al., 2010
tGnd1	*S. cerevisiae*	Nonsense	Continuous	GND1	Constitutive	C-terminus	Miller et al., 2015
DegAB	*S. cerevisiae*)	Degron (contains degradation signal)	Continuous	N/A	Constitutive – TDH3	N-terminus	Shiber et al., 2013
Htt103Q	*H. sapiens*	Huntington’s disease	Continuous	Htt25Q	Induced – GAL	C-terminus	Krobitsch and Lindquist, 2000
β-amyloid	*H. sapiens*	Alzheimer’s disease	Continuous	N/A	Induced – GAL	C-terminus	Treusch et al., 2011
Alpha synuclein	*H. sapiens*	Parkinson’s disease	Continuous	N/A	Induced – GAL	C-terminus	Outeiro and Lindquist, 2003
FUS	*H. sapiens*	Amyotrophic lateral sclerosis (ALS)	Continuous	N/A	Induced – GAL	C-terminus	Fushimi et al., 2011; Kryndushkin et al., 2011; Sun et al., 2011
TDP-43	*H. sapiens*	Amyotrophic lateral sclerosis (ALS)	Continuous	N/A	Induced – GAL	C-terminus	Johnson et al., 2008; Kryndushkin et al., 2011
OPTN	*H. sapiens*	Amyotrophic lateral sclerosis (ALS)	Continuous	N/A	Induced – GAL	C-terminus	Kryndushkin et al., 2012

*References are to the earliest use of the misfolded reporter in the study of spatial quality control.*

## Temperature-Sensitive Misfolding Proteins

### Luciferase, FlucSM/DM

*Photinus pyralis* luciferase was an early model substrate used in the study of PQC. It was selected to elucidate the cellular chaperone machinery in *Escherichia coli* because it was thermolabile, could be reactivated *in vivo*, and activity could readily be monitored by luminescence assay (Schröder et al., 1993). A fluorescently tagged version was later used to study spatial quality control in *E. coli* (Winkler et al., 2010) before versions were adapted for use in *S. cerevisiae* (Specht et al., 2011). Mutant versions of Luciferase, FlucSM/DM were developed to be more susceptible to heat denaturation (Gupta et al., 2011). *S. cerevisiae* compatible constructs were recently developed (Ruan et al., 2017).

### Ubc9ts (ubc9-2)

Temperature-sensitive mutants of *S. cerevisiae* genes have been used to study gene function for decades, particularly of essential genes. Many Ts alleles behave as effective nulls and one mechanism for this was shown with the gene product of a Ts allele of the ubiquitin conjugating enzyme, Ubc9. Several mutant ubc9 proteins were shown to be short-lived at the restrictive temperature and the observed rapid breakdown could be suppressed and was dependent on proteasome activity (Betting and Seufert, 1996). A GFP tagged version of ubc9-2 containing the point mutation Y69L was then used to show that misfolding proteins partition between at least two quality control compartments, the JUNQ and IPOD (Kaganovich et al., 2008).

### guk1-7, gus1-3, pro3-1, ugp1-3

A screen of a panel of 22 Ts alleles of six essential genes encoding predominantly cytoplasmic proteins showed that a significant fraction was degraded at the restrictive temperature, clearly demonstrating degradation as a major mechanism for Ts phenotypes (Khosrow-Khavar et al., 2012). Four unstable mutants were fluorescently tagged and used as model misfolding substrates: guk1-7, gus1-3, pro3-1, and ugp1-3 (Comyn et al., 2016). A Ts mutant of a guanylate kinase, guk1-7, was selected for further characterization. The temperature sensitivity of guk1-7 is a consequence of four missense mutations. It was shown to co-localize with Hsp104-mCherry and with Hsp42-mCherry foci (Comyn et al., 2016), and based on these markers, they are likely deposited into one or more of the major PQCs like Q-bodies, JUNQ/INQ or IPOD.

## Quality Control of Temperature-Sensitive Proteins

Many temperature sensitive proteins are not degraded at the restrictive temperature. The conditional lethal phenotype caused by these stable variants are likely due to local perturbations in domain structure caused by the mutations. This local effect is apparent in Ts alleles that encode homomultimeric proteins where intragenic complementation is possible (Sundberg and Davis, 1997). Dominant Ts alleles have been proposed to affect protein structure locally (McMurray, 2014). Indeed, these local effects may generally help to explain the wide range of phenotypes that can be observed with different Ts mutants of the same gene.

In contrast, a significant percentage of temperature-sensitive mutants display a recessive, null phenotype at the restrictive temperature that is often due to degradation of the expressed protein (Khosrow-Khavar et al., 2012). The null phenotype of unstable Ts alleles can often be rescued by removal of one or a combination of genes involved in PQC, indicating that the mutations do not significantly affect protein function, but instead cause partial unfolding that the quality control machinery recognizes, resulting in that protein being targeted for destruction (Betting and Seufert, 1996; Gardner et al., 2005; Khosrow-Khavar et al., 2012). It is this class of Ts mutants that has been adapted for the study of PQC.

Of the Ts substrates adapted, Luciferase is the only one with several well-established assays to monitor enzymatic activity, which is one of the main reasons it was initially chosen as a model substrate (Schröder et al., 1993). When used as a spatial quality control substrate, it has a rather mild aggregation phenotype that either requires a higher 42°C heat shock or proteasomal inhibition for aggregates to be clearly visible. As a consequence, destabilized versions of luciferase, FlucSM and FlucDM were later engineered to make them more amenable to studies of proteome stress (Gupta et al., 2011). FlucSM expressed in yeast forms clear aggregates at 42°C, but experiments with milder 37°C heat stress have not yet been reported in yeast (Ruan et al., 2017). Luciferase is also the only exogenous Ts substrate used in the study of spatial quality control. This has the potential advantage that it is unlikely to specifically interact with endogenous proteins. However, it may also not be recognized by the PQC machinery in the same way as an endogenous protein, potentially limiting its use in elucidating endogenous spatial quality control pathways.

The remaining substrates are Ts versions of *S. cerevisiae* proteins which have been selected to be unstable at or above 37°C. Ubc9-2 has often been used as a spatial quality control substrate since it was originally used to help define the JUNQ and IPOD deposition sites (Kaganovich et al., 2008). The guk1-7, gus1-3, pro3-1, and ugp1-3 proteins expand the number of available Ts substrates significantly and allow interesting comparisons between processing of endogenous misfolded proteins since they differ in their wild-type gene function, cellular localization and pathways of degradation upon misfolding. At the permissive temperature, these Ts substrates have been reported to have the following localization patterns: ubc9-2, nucleus; guk1-7, nucleus and cytoplasm; gus1-3, cytoplasm and mitochondria; pro3-1, cytoplasm; ugp1-3, cytoplasm and plasma membrane^1^. It is perhaps not surprising that these model proteins show notable differences in the way they are processed by the PQC machinery. For example, degradation of pro3-1 is partially dependent on the E3 ubiquitin ligase San1 for degradation while guk1-7 is significantly less dependent. They also show different responses to deletion of prefoldin subunits. Furthermore, gus1-3 appears to be unique in that it does not appear to depend on the proteasome for degradation (Khosrow-Khavar et al., 2012). **Table 2** summarizes what is known about the processing of the Ts model substrates.

**Table 2:** Spatial quality control pathways implicated in processing of the misfolding model substrates, as well as dependence on the proteasome for degradation.

Name	Degradation	Ubr1 dependent?	San1 dependent?	Sorted to JUNQ/INQ?	Sorted to IPOD?	Reference
Luciferase	Proteasome	Yes	N/D	Yes	No	Nillegoda et al., 2010; Miller et al., 2015
FlucSM/DM	Proteasome	N/D	N/D	N/D	N/D	Gupta et al., 2011; Ruan et al., 2017
ubc9-2	Proteasome	N/D	N/D	Yes	Yes	Betting and Seufert, 1996; Kaganovich et al., 2008
guk1-7	Proteasome	Yes	No	Yes^1^	N/D	Khosrow-Khavar et al., 2012; Comyn et al., 2016
gus1-3	Unknown	N/D	N/D	N/D	N/D	Khosrow-Khavar et al., 2012
pro3-1	Proteasome	Yes	Yes	N/D	N/D	Khosrow-Khavar et al., 2012
ugp1-3	Proteasome	Yes	N/D	N/D	N/D	Khosrow-Khavar et al., 2012
Actin(E364K)	Proteasome	N/D	N/D	Yes	Yes	Kaganovich et al., 2008
VHL	Proteasome	N/D	N/D	Yes	Yes	McClellan et al., 2005; Kaganovich et al., 2008
ΔssCPY^∗^	Proteasome	Yes	Yes	Yes	Yes^1^	Eisele and Wolf, 2008; Miller et al., 2015
ΔssPrA	Proteasome	No	Yes	Yes^1^	Yes^1^	Prasad et al., 2010
tGnd1	Proteasome	Yes	Yes	Yes	Yes^1^	Heck et al., 2010; Miller et al., 2015
DegAB	Proteasome	N/D	N/D	Yes	Yes^1^	Furth et al., 2011; Alfassy et al., 2013; Shiber et al., 2013, 2014
Htt103Q	Proteasome, autophagy	N/D	N/D	No	Yes	Kaganovich et al., 2008; Chuang et al., 2016
β-amyloid	Secretory pathway	N/D	N/D	N/D	N/D	Treusch et al., 2011; D’Angelo et al., 2013
Alpha synuclein	Proteasome, autophagy	N/D	N/D	No	No	Outeiro and Lindquist, 2003; Petroi et al., 2012; Tenreiro et al., 2014
FUS	N/D	N/D	N/D	No	Yes, only N-terminal fusion	Kryndushkin et al., 2012
TDP-43	Proteasome, autophagy	N/D	N/D	No	No	Farrawell et al., 2015; Leibiger et al., 2018
OPTN	Proteasome	N/D	N/D	No	Partially	Kryndushkin et al., 2012

*N/D, not determined. ^1^inferred from relative location*.

**FIGURE 2:**
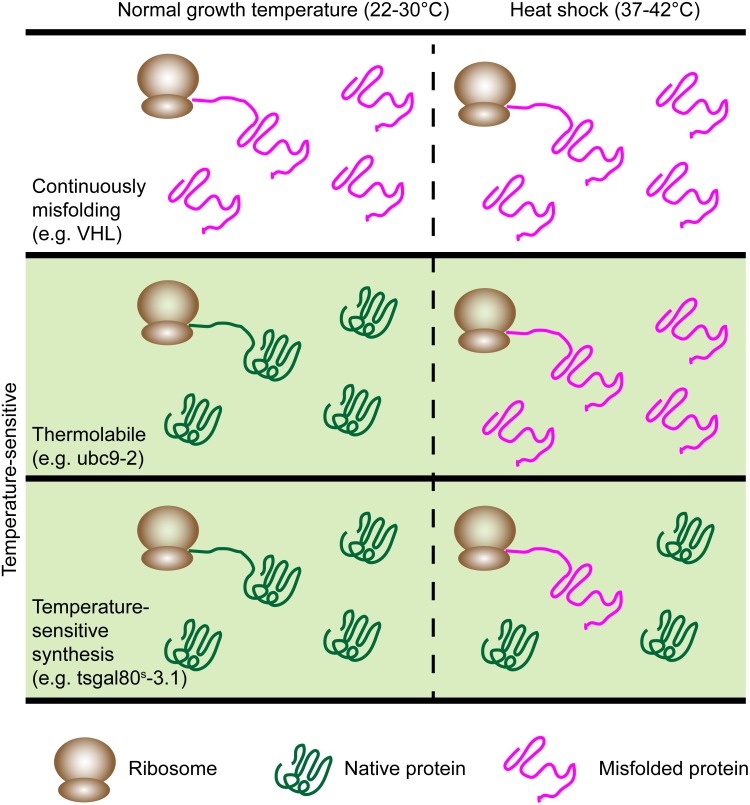
The model proteins have different patterns of misfolding. Continuously misfolding proteins misfold at all temperatures. Ts proteins (green panel) misfold during heat shock and fall into two classes: Thermolabile (TL) and temperature-sensitive synthesis (TSS). TL proteins universally misfold during heat shock while TSS proteins misfold only if synthesized during heat shock. Examples are listed for each category.

There are further subclasses of Ts proteins that are important to distinguish between, especially in the context of quality control. Some proteins are TL (thermolabile) while others are TSS (temperature sensitive synthesis) (Sadler and Novick, 1965; McMurray, 2014) (**Figure 2**). TL mutants are universally destabilized at the restrictive temperature, whereas TSS mutants are those that only misfold during synthesis. In yeast, a clear distinction between these phenotypes was shown with Ts mutants of Gal80 (Matsumoto et al., 1978). These unique properties can be exploited to examine whether the PQC machinery handles misfolding of newly synthesized or aged proteins differently. Of the Ts proteins used to study spatial quality control, only Ubc9ts has been characterized in this way and was shown to be TL as it could form foci during heat shock even after expression was shut off (Escusa-Toret et al., 2013). For this reason, we cannot generally distinguish between TL and TSS in this review.

## Continuously Misfolding Proteins

### Actin(E364K)

The actin mutant actin(E364K) was originally isolated in *Drosophila melanogaster* (Drummond et al., 1991) and was later shown to be degraded by the PQC machinery (McClellan et al., 2005). A GFP tagged version localized to quality control compartments seen with ubc9-2 (Kaganovich et al., 2008).

### von Hippel–Lindau (VHL)

The von Hippel–Lindau (VHL) tumor-suppressor protein is subject to chaperone mediated folding in mammalian cells (Feldman et al., 1999). Tumor-causing mutations that disrupt VHL binding to Elongin B/C leads to misfolding and degradation by the proteasome (Feldman et al., 1999; Schoenfeld et al., 2000). Wild type VHL was shown to be degraded in yeast due to absence of Elongin B/C and this was similarly dependent on the ubiquitin-proteasome pathway (McClellan et al., 2005). GFP tagged VHL was shown to be subject to spatial quality control in yeast similar to ubc9-2 and actin(E346K) (Kaganovich et al., 2008).

### ΔssCPY^∗^

The first mutant version of the vacuolar enzyme carboxypeptidase Y (Y, yscY, CPY, PRC1) was isolated in 1975 (Wolf and Fink, 1975) in a screen with the purpose of investigating the function of the proteinase itself. However, CPY^∗^ did not localize to the vacuole, its regular destination, but instead was retained in the ER for proteasome-independent rapid degradation and was found to be misfolded (Finger et al., 1993).

Many derivatives of CPY have been used to study their degradation or PQC pathways, which differ depending on the domains they are fused to, e.g., the fusion to a transmembrane domain to study ER membrane proteins (Stolz and Wolf, 2012). One such derivative is ΔssCPY^∗^, which is a cytoplasmic misfolding substrate made by eliminating the ER-targeting signal sequence from CPY^∗^. Though originally used as a negative control when studying ERAD dependent degradation, (Medicherla et al., 2004) GFP tagged ΔssCPY^∗^ was shown to form inclusions and is subject to spatial quality control (Prasad et al., 2010; Park et al., 2013). Based on this construct, a similar cytosolic misfolding protein, ΔssPrA, was made using vacuolar proteinase A (PEP4) (Prasad et al., 2010).

### tGnd1

A dosage suppressor screen using a high copy (2 μ) genomic library was performed to identify factors involved in stabilization of the misfolded model ΔssCPY^∗^ (Heck et al., 2010). The recovered hits were found to express truncated proteins, one of which was a truncated form of Gnd1, a phosphogluconate dehydrogenase. The truncation proved to be a competing PQC substrate. Fluorescently tagged tGnd1 was shown to localize to JUNQ/INQ and CytoQ deposits (Miller et al., 2015).

### DegAB

A study of the degradation signal (degron) of the kinetochore protein, Ndc10, showed that it functions autonomously, as it leads to degradation of various other stable proteins when it is attached (Furth et al., 2011). This degron consists of two parts, DegA and DegB, leading to the collective name DegAB (Alfassy et al., 2013). This model degron differs from conventionally used terminally misfolding model substrates for PQC as it does not aggregate spontaneously, is not cytotoxic and can model mildly misfolded PQC substrates, which remain soluble and derive from an endogenous yeast protein (Shiber et al., 2013). A GFP fusion of DegAB, GFP-DegAB, can be used to study spatial PQC and generally forms two inclusions, one of which is juxtanuclear.

## Quality Control of Continuously Misfolding Proteins

All non-thermolabile misfolding proteins presented here are targeted for degradation via the ubiquitin-proteasome pathway (Schoenfeld et al., 2000; McClellan et al., 2005; Park et al., 2007; Heck et al., 2010; Furth et al., 2011). Nonetheless, the factors required for degradation of substrates differ. For example, actin(E364K) and VHL have been compared in the same study and it was shown that Hsp90 is required for VHL degradation but not for actin(E364K) degradation. Additionally, VHL requires the Hsp70 chaperone Ssa1 and its co-chaperone Sti1 for degradation (McClellan et al., 2005). ΔssCPY^∗^ and tGnd1 share a requirement for the Ubr1 and San1 E3 ubiquitin ligases for their degradation (Eisele and Wolf, 2008; Heck et al., 2010; Miller et al., 2015). Similarly, DegAB was found to depend on Ssa1 or Ssa2 (Shiber et al., 2013).

Another similarity of all non-thermolabile substrates studied so far in this regard is their spatial sorting in the cell, as they have been observed to at least partially localize to both the JUNQ/INQ and IPOD compartment upon aggregation. Actin(E364K) and VHL both localize to JUNQ/INQ and IPOD but VHL requires heat shock for localization to the IPOD (Kaganovich et al., 2008). Proteasome inhibition alone results in a single nuclear inclusion, most likely the JUNQ/INQ (Kaganovich et al., 2008; Specht et al., 2011; Spokoini et al., 2012; Miller et al., 2015). ΔssCPY^∗^ and tGnd1 have been demonstrated to localize to the JUNQ/INQ in the stabilizing ubr1Δ san1Δ background in which these model proteins are no longer, or only marginally, ubiquitinated. This observation lead to the conclusion that ubiquitination is not a requirement for JUNQ/INQ targeting (Miller et al., 2015). In these experiments, tGnd1 is also targeted to a second cytoplasmic focus, which most likely corresponds to the IPOD, while ΔssCPY^∗^ appears exclusively targeted to INQ. However, cytoplasmic foci have also been observed for ΔssCPY^∗^. DegAB foci were shown to co-localize with Hsp42 and Hsp104 (Shiber et al., 2013) and detergent solubility properties of DegAB were determined to be similar to those of ΔssCPY^∗^ as measured by flow cytometry (Shiber et al., 2014). In summary, there are clear differences in sorting of these model proteins, which makes it reasonable to use more than one misfolding substrate when general conclusions about spatial sorting pathways are to be drawn.

## Human Disease Proteins

Age-associated proteopathies have been increasingly modeled in yeast to gain more nuanced insight into the molecular bases of the diseases while circumventing potential ethical issues such as those with patient samples. The successful introduction of a range of different disease model proteins highlights the versatility and applicability of this model organism in the study of proteostasis, aging, and disease development (Braun et al., 2010). Different types of high-throughput screens with budding yeast are particularly powerful and have been used, in part, to find modifiers of cytotoxicity of disease proteins (Willingham et al., 2003; Cooper et al., 2006; Liang et al., 2008; Elden et al., 2010; Ju et al., 2011; Kayatekin et al., 2014). Importantly, fluorescently tagged versions of several proteins implicated in disease progression have been introduced to study how the spatial quality control machinery handles them and to help determine what contributes to their toxicity in diseased neurons. Here, we focus exclusively on substrates that have been shown to be subject to spatial quality control and aim to provide guidance in the selection of model substrates used to study spatial quality control pathways relevant to these proteopathies. For more thoroughly detailed descriptions of yeast models of human neurodegenerative disease, we refer the reader several excellent reviews (Braun et al., 2010; Pereira et al., 2012; Tenreiro et al., 2013; Braun, 2015; Menezes et al., 2015; Fruhmann et al., 2017). The disease models are summarized below and common constructs are listed in **Table 1**.

### Huntington’s Disease

#### Htt103Q/Htt97Q

Yeast models for characterization of Huntingtin (Htt), the protein that is mutated in patients of Huntington’s disease, were established by testing variants of exon 1 of the N-terminus of human Huntingtin. These differ in the lengths of the polyglutamine expansions that modify aggregation behavior (Krobitsch and Lindquist, 2000). The Huntingtin model protein commonly used in yeast is Htt103Q, which is named after the number of glutamines in the expansion. A non-expanded variant, Htt25Q, is used as a control. Non-expanded variants, like Htt25Q, display diffuse localization in the cytoplasm, while expansions of 72 glutamines or more cause the proteins to form visible aggregates of varying size and quality (Krobitsch and Lindquist, 2000). Interestingly, it has been reported that an intermediate length huntingtin with a 47 glutamine expansion forms aggregates in yeast upon entry into stationary phase, which supports the use of stationary phase cells as a reasonable model for aged neuronal cells (Cohen et al., 2012). Models with longer expansions, such as Htt103Q, have different patterns of localization, ranging from on major focus per cell to multiple foci, that depend on expression levels and the regions flanking the polyglutamine expansion (Krobitsch and Lindquist, 2000; Duennwald et al., 2006; Wang et al., 2009; Song et al., 2014; Berglund et al., 2017).

### Alzheimer’s Disease

#### β-Amyloid

Alzheimer’s disease (AD) models focus on one of the proteins implicated in the pathology of AD, the amyloid-β peptide (Aβ), as extracellular amyloid plaques containing Aβ appear in affected individuals. The Aβ peptides arise through cleavage of the amyloid precursor protein (APP) via the amyloidogenic pathway, resulting in different sizes of Aβ, mostly Aβ_40_ and Aβ_42_ (O’Brien and Wong, 2011). The latter is more aggregation-prone and nucleates plaques in diseased individuals and is therefore often selected as a model. A GFP-Aβ_42_ fusion protein was shown to form aggregates and induced a stress response (Caine et al., 2007). Toxicity models expressing the Aβ peptide were established later by targeting Aβ to the secretory pathway (Treusch et al., 2011; D’Angelo et al., 2013). Expression via an inducible promoter caused cytotoxicity without extensive cell death compared to a less toxic Aβ_40_ peptide control (Treusch et al., 2011).

### Parkinson’s Disease

#### Alpha Synuclein

Alpha synuclein (aSyn) is a lipid-binding protein and the main constituent of Lewy Bodies, which are protein deposits occurring in diseased neurons that are affected by Parkinson’s Disease (PD) and other disorders. aSyn and derivatives were first investigated in mammalian cells (McLean et al., 2001) and later used to develop a PD model in yeast (Outeiro and Lindquist, 2003). While several PD models have been established in yeast [reviewed in (Menezes et al., 2015)], aSyn is the most commonly used model protein. Expression of aSyn in yeast causes dose-dependent cytotoxicity, a phenotype reminiscent of what is seen in human cells. Toxicity can therefore be modified by inducing different levels of expression by regulatable promoters or by changing plasmid copy number. As with human cells, aSyn has been reported to localize mainly to the plasma membrane and partially to the cytoplasm. However, overexpression causes visible aggregation of aSyn into cytosolic inclusions (Outeiro and Lindquist, 2003).

### Amyotrophic Lateral Sclerosis (ALS)

#### FUS, TDP-43, OPTN

Mutations associated with amyotrophic lateral sclerosis (ALS) appear in the proteins SOD1, FUS, TDP-43, and OPTN which become misfolded and aggregate. Yeast studies have focused on FUS and especially TDP-43. Both proteins act as DNA/RNA-binding proteins and are found in neuronal inclusions of affected individuals. A TDP-43 model in yeast mimics several disease characteristics. Expression from an inducible plasmid causes it to form nuclear inclusions, while higher expression induces mislocalization to the cytoplasm where it forms visible inclusions (Johnson et al., 2008). These cytoplasmic aggregates are toxic to the cell. Similarly, FUS models have shown that expression in yeast is also cytotoxic (Fushimi et al., 2011; Kryndushkin et al., 2011; Sun et al., 2011). The model protein forms numerous cytoplasmic aggregates, which co-localize with and cause formation of RNA processing sites (P-bodies and stress granules) as observed in human cells (Kryndushkin et al., 2011). Another protein known to form inclusions in ALS-affected individuals is optineurin (OPTN). In a recently developed yeast model, wild type OPTN was shown to be toxic, as were versions with disease-causing mutations (Kryndushkin et al., 2012).

## Quality Control of Human Disease Proteins

The disease proteins summarized above do not require stress conditions such as heat shock or proteasome inhibition to induce aggregate formation since they aggregate autonomously. Therefore, they are most commonly expressed using inducible promoters (**Table 1**). Additionally, it should be noted that none of the human misfolding proteins discussed here have orthologs in yeast.

The spatial quality control mechanisms handling several disease model proteins in yeast have not yet been extensively studied, however, it appears that amyloidogenic proteins in general are targeted to the IPOD compartment, which has been shown using Htt mutant proteins (Kaganovich et al., 2008; Escusa-Toret et al., 2013). The aggregates formed by aSyn are likely neither localized to JUNQ nor IPOD, as they did not co-localize with any of the commonly used markers for these quality control compartments (Outeiro and Lindquist, 2003; Tenreiro et al., 2014). In contrast to the other disease model proteins, TDP-43, FUS, and OPTN form non-amyloid aggregates. The spatial quality control mechanisms involved in the transport and processing of TDP-43, FUS, and OPTN have not been extensively investigated in yeast. A study using a mouse cell line concluded that the inclusions visible for TDP-43 and FUS might be distinct from several of the known quality control compartments: aggresome, JUNQ and IPOD (Farrawell et al., 2015). However, both TDP-43 and FUS have been reported to co-localize and physically interact in yeast (Kryndushkin et al., 2011), suggesting involvement of similar spatial PQC mechanisms for both model proteins. Furthermore, a GFP tagged version of FUS localizes to the IPOD while OPTN also shows only partial localization to this quality control site (Kryndushkin et al., 2012). OPTN forms non-amyloid cytoplasmic aggregates, which are distinct from FUS and TDP-43 foci, as they do not exclusively co-localize. OPTN appears to be handled in a unique way by the cell in that a single focus appears early after induction of expression, while upon later time points several small additional foci become visible. The foci only partially overlap with previously characterized model proteins reported to localize to the IPOD, further indicating that additional deposition sites may exist in the cytoplasm.

## General Considerations for Model Selection

When selecting a candidate model misfolding protein, it is useful to consider what type of misfolding is to be modeled, the temperature sensitivity, whether a properly folding control is available, the promoter used for expression and the effects of the fluorescent tag. The categories are listed in **Table 1** and summarized below.

### Yeast vs. Non-yeast

Both mutated yeast proteins and non-yeast proteins have been used to study spatial quality control in *S. cerevisiae*. Use of mutated native proteins, such as the frequently used Ubc9ts, has the general advantage that it most closely mimics a typical error during protein production. The yeast spatial PQC machinery is more likely to properly recognize a misfolded endogenous protein compared to a non-native protein and it is therefore more likely to be sequestered to an “authentic” deposition site in the cell. However, mutated yeast proteins may also cause dominant negative effects, particularly when overexpressed, for example by sequestration of native binding partners. Non-native proteins, particularly those lacking yeast homologs, are less likely to bind endogenous proteins and can therefore be useful alternatives in this regard.

### Mutant Model

Aberrant folding of proteins in wild-type cells can happen for several reasons. It is a major consequence of stress, such as with heat-induced denaturation. It can also be a consequence of mutations or a result of errors during protein production. Additionally, changes in expression can also result in a lack of binding partners necessary for proper folding. Model misfolding proteins have been engineered or selected to mimic the misfolding that occurs in these instances so that specific responses by the PQC machinery can be elucidated.

### Temperature-Sensitive vs. Continuously Misfolding Reporters

Ts substrates are useful as unfolding can be triggered easily and rapidly by temperature shift so that the immediate response to unfolded proteins can be studied. In the case of TL substrates, it also allows decoupling from translation as one can terminate translation using cycloheximide before temperature shift. However, this temperature shift will also trigger the general heat shock response, which upregulates a range of chaperones. Non-thermolabile proteins do not require this temperature shift; therefore, while chaperones may be induced in response to the continuously misfolding substrate, temperature-induced changes in the levels of molecular chaperones are avoided.

### Normally Folding Controls

Negative controls play an important role in studies on PQC as it is important to assess whether observable effects on PQC can be actually attributed to the misfolding protein itself and not to other factors such as their fluorescent tag or dominant negative effects. Negative controls exist for a subset of the human disease models. The Huntingtin models expressed in yeast, Htt103Q or other variants with expanded polyQ stretches, can be compared to a diffusely distributed cytosolic version, Htt25Q, which serves as a wild type control. Similarly, there are comparably less toxic variants of the amyloid beta peptide, which can be used as controls in some experimental setups. Alpha-synuclein aggregation depends on the level of expressed protein. While high expression levels result in aggregation, expression from a low copy number plasmid leaves the protein localized to the plasma membrane and the cytoplasm. A similar principle applies to TDP-43.

Several misfolding proteins can be directly compared to a wild-type control. For example, Ubc9 is used as a control for ubc9-2 (Kaganovich et al., 2008). Corresponding wild-type alleles are also available for guk1-7, gus1-3, pro3-1, and ugp1-3 (Khosrow-Khavar et al., 2012). Wild-type Gnd1 is used as a control for the truncated misfolding tGnd1 (Heck et al., 2010). However, the majority of the continuously misfolding proteins including VHL, actin(E364K), ΔssCPY^∗^, and DegAB do not have obvious normally folding controls.

### Promoter

Misfolding substrates have generally been placed behind strong promoters, such as TDH3, with the intent of overloading the temporal quality control system in order to easily visualize aggregates and inclusions. If the model protein of choice is not obviously cytotoxic, constitutively active promoters are useful in that they require no change in experimental conditions (such as a change in carbon source) to induce expression.

Inducible promoters have been commonly used when working with misfolding proteins that are cytotoxic, such as alpha-synuclein (**Table 1**). Thereby, cytotoxic effects of the misfolding protein can be avoided during strain construction and limited to the experiment itself. Additionally, inducible promoters can be useful for misfolding proteins that show a dose-dependent cytotoxicity, as it is easy to compare cytotoxicity when the expression of the misfolding protein is tightly regulated. It is also possible to shut off translation of the misfolding protein at a specific time point without interfering with translation of other proteins with agents such as cycloheximide. This is especially important when the cellular response to any particular misfolding protein is partially or fully dependent on a functional translation machinery.

One major limitation of the constitutive and inducible promoters used for the vast majority of the models covered in this review, is that they are among the strongest promoters characterized in *S. cerevisiae* (Peng et al., 2015). While inclusions can easily be visualized as a result, the high concentration of protein can cause toxicity unrelated to protein misfolding. For example, it can lead to sequestration of native and non-native binding partners and it may result in transport into non-native cellular compartments or organelles. Normally folding controls may mitigate this problem, as they can be used to identify the abnormal or toxic effects of overexpression.

### Fluorescent Tagging

Fluorescent tags should, ideally, minimally affect protein function and are therefore generally placed accordingly. Practically, however, the tag will always have some effect regardless of whether it is placed N-terminally, C-terminally, or at an internal site. Even though the proteasome can process N-terminally or C-terminally tagged substrates (Liu et al., 2003), tagging can stabilize certain mutants (Mikalsen et al., 2005; Khosrow-Khavar et al., 2012), perhaps due to shielding of the unfolded domain recognized by the quality control machinery. Tag location will also determine if the misfolding protein exits the ribosome before or after folding of the fluorescent tag can begin, which can affect stability of the model protein or have an effect on the fluorescent tag itself. In fact, certain misfolded proteins have been shown to significantly affect GFP chromophore formation. This effect was taken advantage of to develop a protein folding assay (Waldo et al., 1999). In this study, fluorescence of GFP tagged test substrates correlated with solubility, indicating that aggregation of their test substrates affected chromophore formation. Therefore, for any substrate, it is important to determine whether the fluorescent signal observed is proportional to the amount of protein present. Along the same lines, the fluorescent signal may not reflect the presence of a misfolded protein as the proteasome can degrade it while leaving the fluorophore intact (Liu et al., 2003). Finally, the tag itself has been shown to influence toxicity of misfolding proteins. Tagging affected toxicity of different huntingtin constructs (Jiang et al., 2017) and the location of the GFP-tag and the design of the linker on FUS and OPTN was shown to influence toxicity and the constructs’ aggregation propensities (Kryndushkin et al., 2012).

## Concluding Remarks

Deposition into quality control sites is a common, if not universal, response to misfolding proteins as these sites have been described in a wide range of organisms. Model misfolding proteins have been key tools used in the identification of, and distinction between, these quality control sites. Importantly, the use and characterization of a range of different misfolded proteins in yeast has made it clear that while many are handled similarly, there are often notable differences. For example, several models vary in their dependence on the San1 and Ubr1 E3 ubiquitin ligases for proteasomal degradation, while others do not depend on the proteasome but are degraded or removed in an undefined manner (**Table 2**). Furthermore, human disease models appear to be even more distinct in their processing. The huntingtin model, along with other amyloid aggregates, does not obviously sort to the JUNQ deposition site and ALS model proteins appear to deposit at sites that are distinct from both the JUNQ and IPOD. Similarly, aggregates of the Parkinson’s disease model, aSyn, did not colocalize to these defined sites (Tenreiro et al., 2014). Taken together, the evidence points toward the existence of uncharacterized spatial quality control pathways or, at the very least, modifications of defined pathways that have not yet been well characterized. Continued use of model misfolding proteins under varying conditions alone, and in combination, will do much to further define these spatial quality control pathways that play such an important role in the maintenance of proteostasis and prevention of disease progression.

## Author Contributions

PW designed the review outline. PW and KS wrote the manuscript with guidance and input from TN.

## Conflict of Interest Statement

The authors declare that the research was conducted in the absence of any commercial or financial relationships that could be construed as a potential conflict of interest.

## Footnotes

1 https://www.yeastgenome.org/
